# Pallidal Index as Biomarker of Manganese Brain Accumulation and Associated with Manganese Levels in Blood: A Meta-Analysis

**DOI:** 10.1371/journal.pone.0093900

**Published:** 2014-04-09

**Authors:** Shao-Jun Li, Li Jiang, Xue Fu, Shuang Huang, Yan-Ni Huang, Xiang-Rong Li, Jing-Wen Chen, Yong Li, Hai-Lan Luo, Fang Wang, Shi-Yan Ou, Yue-Ming Jiang

**Affiliations:** 1 Department of Toxicology, School of Public Health, Guangxi Medical University, Nanning, Guangxi, China; 2 Department of Radiotherapy, the First Affiliated Hospital of Guangxi Medical University, Nanning, Guangxi, China; 3 School of Health Sciences, Purdue University, West Lafayette, Indiana, United States of America; 4 Department of Radiology, the First Affiliated Hospital of Guangxi Medical University, Nanning, Guangxi, China; The Pennsylvania State University Hershey Medical Center, United States of America

## Abstract

**Objective:**

The current study was designed to evaluate the sensitivity, feasibility, and effectiveness of the pallidal index (PI) serving as a biomarker of brain manganese (Mn) accumulation, which would be used as an early diagnosis criteria for Mn neurotoxicity.

**Methods:**

The weighted mean difference (WMD) of the PI between control and Mn-exposed groups was estimated by using a random-effects or fixed-effects meta-analysis with 95% confidence interval (CI) performed by STATA software version 12.1. Moreover, the R package “metacor” was used to estimate correlation coefficients between PI and blood Mn (MnB).

**Results:**

A total of eight studies with 281 occupationally Mn-exposed workers met the inclusion criteria. Results were pooled and performed with the Meta-analysis. Our data indicated that the PI of the exposed group was significantly higher than that of the control (WMD: 7.76; 95% CI: 4.86, 10.65; *I^2^* = 85.7%, *p*<0.0001). A random effects model was used to perform meta-analysis. These findings were remarkably robust in the sensitivity analysis, and publication bias was shown in the included studies. Seven out of the eight studies reported the Pearson correlation (r) values. Significantly positive correlation between PI and MnB was observed (r = 0.42; 95% CI, 0.31, 0.52).

**Conclusions:**

PI can be considered as a sensitive, feasible, effective and semi-quantitative index in evaluating brain Mn accumulation. MnB can also augment the evaluation of brain Mn accumulation levels in the near future. However, the results should be interpreted with caution.

## Introduction

Manganese (Mn),naturally exists in rocks, soil, water and food, is an essential trace element for human beings [1]–[3]. In the central nervous system (CNS), Mn serves as a cofactor for key enzymes such as astrocytic glutamine synthetase, pyruvate carboxylase, and mitochondrial superoxide dismutase [4]–[6]. However, exposure to excess Mn can cause an extrapyramidal syndrome known as manganism [7], which shows similar dystonic movements to the Parkinson's disease (PD). Mn can be transported across the blood-brain barrier and selectively accumulate in basal ganglia, putamen, substantia nigra and anterior pituitary, particularly in the globus pallidus (GP), resulting in serious and irreversible neurological disorders [6]. Thus, by monitoring the deposition level of Mn in the brain may provide observation, evaluation, and intervention of early neurotoxic effects of Mn.

Magnetic resonance imaging (MRI) technology has taken the advantages of the paramagnetic properties of Mn compound identified as a potential indirect, noninvasive contrast marker [8]–[10] and the identification of neuronal pathways through rodent brains in tract tracing studies [11]. Brain MRI showed T_1_-weighted intensity (T_1_-WI) abnormal signal enhancement in the striatum, GP and substantia nigra in Mn-exposed animals [12]–[14] and asymptomatic occupationally Mn-exposed workers [15], while no alteration in the T_2_-WI signal was found. In addition, significantly increased T_1_-WI signal intensities in the GP were observed in patients with chronic liver diseases [16], [17], patients receiving total parenteral nutrition [18], [19], Wilson's disease and Rendu-Osler-Weber disease[20], [21], suggesting that MRI T_1_-WI may be used as a non-invasive examination to detect Mn accumulation in the brain.

Many valuable studies have been published on the application of MRI to detect brain Mn accumulation, but there is no unified and systematic evaluation criterion. Previous researchers have used the pallidal index (PI) as a semi-quantitative parameter to evaluate the Mn accumulation in the brain, which is calculated as the signal intensity ratio of GP relative to the frontal white matter (FWM) on T_1_-WI [22], [23]. Although PI is the only indicator for brain Mn deposit level, it is fraught with potential for subjective assessment. The signal intensities of GP and FWM are detected by subjectively selecting regions of interest (ROI) chosen by the operators using a computer [22]. Furthermore, the relationship between PI and brain Mn accumulation and blood Mn (MnB), and whether the Mn level in the brain is a result of early neurotoxic effect that can be reflected by PI, remain uncertain. Therefore, given the diverse findings in previous Mn studies, we performed a meta-analysis of PI and correlation coefficients between PI and MnB by pooling data from the current literatures that used MRI to detect Mn accumulation in the brain Mn-exposed workers, in order to assess the sensitivity, feasibility and effectiveness of PI as a semi-quantitative evaluation indicator for the evaluation of Mn levels in the brain.

## Materials and Methods

### Inclusion criteria

All of the included studies were required to meet the following inclusion criteria:

1. The study must be a retrospective study, clinical trial or quasi-randomized controlled trial.
2. The objects of study
  The control group of the study was comprised of the healthy subjects without exposure to Mn or Mn-dioxide in a work environment with Mn concentrations exceeding 0.15 mg/m^3^ of the time-weighted average allowable concentration, as prescribed by the “Harmful factors of workplace occupationally exposure limits”. Subjects in the experimental group were occupational exposure to Mn for a work period of 8 h/d, 5 d/wk.
3. The MRI examination and PI calculation of the study
  MRI examinations were performed using a model of whole body scanner equal to or better than 0.5T (Tesla, magnetic induction units) for both control and experimental groups. PI was calculated, with PI defined as the ratio of the signal intensity of the GP to that of the sub-cortical frontal white matter on axial T_1_-weighted 3D FSPGR MR imaging, multiplied by 100.
4. Means and standard deviations of the PI of the experimental and control groups or Pearson correlation (r) values of PI and MnB were reported or provided by the investigators upon request.
5. When studies shared common sample sources, only results of the larger study were included to ensure the independence of datasets.

### Exclusion criteria

Studies published only as abstracts, reviews or non-original reports (unless containing new data), duplicate publications of the same dataset, those we could not access full texts despite contacting the author and those that did not meet the inclusion criteria were excluded.

### Search sources and search strategy

Two investigators independently selected and reviewed eligible studies. We searched the following databases, websites and other sources from inception through April 2013: MEDLINE, Cochrane Library, Embase, Chinese Biological Medical Literature (CBM), Chinese National Knowledge Infrastructure (CNKI), Chinese Wanfang, and Chongqing VIP databases for epidemiological studies. References of relevant studies were also screened for eligibility. Additionally, we manually retrieved or wrote to authors to ask for unpublished or more complete information. The search strategy for MEDLINE (via Pub Med) is presented in Table1. This strategy was adapted for searching other databases.

**Table 1:** The search strategy for MEDLINE.

Number	Search expression
#6	#1 AND #2 AND #3 AND #4 AND #5
#5	manganese exposure or occupational manganese exposure
#4	globus pallidus
#3	T_1_-weighted image or T_1_-weighted signal
#2	MRI or magnetic resonance imaging
#1	manganese

Note: #6 indicates a search of the literature for “use MRI detects occupational Mn-exposed caused brain Mn accumulation”.

### Data extraction

We created a data extraction form to assemble previously defined relevant information from the studies, including: a. the general information of included studies (e.g. number, publication year, country, etc.); b. study characteristics (e.g. sample size, average age, trade or craft, etc.); c. the intervention of included studies (e.g. brain Mn accumulation detection technology, equipment specifications, computational formula, etc.); d. outcome variables (e.g. The number of MRI T_1_-WI signal enhancement, the mean and standard deviation of PI, Pearson correlation coefficient and sample size of PI and MnB). Data extraction was performed independently by two investigators and any disagreements were resolved by consensus between the authors. We contacted studies' corresponding authors to obtain missing data not stated in the reports.

### Statistical Analysis

Statistical analyses were conducted using the STATA software version 12.0 and the R package “metacor”. We assessed the strength of association between PI and brain Mn accumulation by estimating the weighted mean difference (WMD) along with the corresponding 95% confidence intervals (CI) and used forest plot analysis on the results which was conducted using STATA software version 12.0. The relationship between PI and MnB was estimated by using correlation coefficients as the effect size performed using the R package “metacor”. Heterogeneity between studies was evaluated using the Q statistic; the *I^2^* index was used to estimate the percentage of variation across studies due to heterogeneity [24]. A random effects model was used if significant heterogeneity (*I^2^*>50%, *p*<0.1) was observed between studies; Otherwise, a fixed effects model was adopted. Sensitivity analysis was performed by removing each study in the meta-analysis one at a time to detect its influence on pooled OR. We explored the potential sources of heterogeneity by meta-regression analysis. Funnel plots and Egger's test were used to assess publication bias.

## Results

### Study Selection

**Figure 1:**
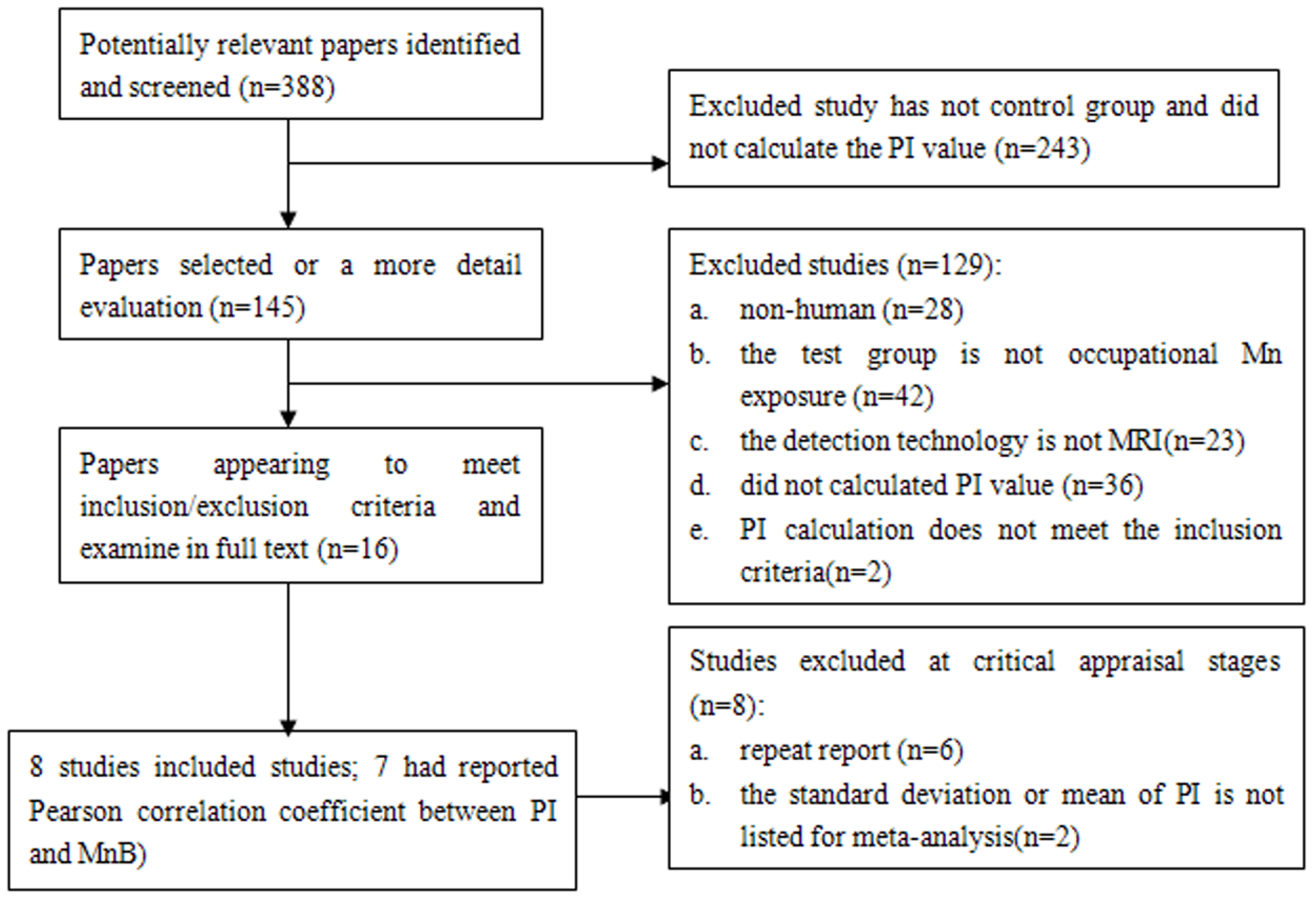
Results of search, selection and inclusion of studies in the review.

Our literature search identified 388 relevant articles. After screening the titles and abstracts, 145 were selected for full text assessment. Of these 145 studies, 129 were excluded: 28 were non-human studies, experimental subjects of 42 studies were not Mn-exposed workers, 23 had not used MRI technology, 36 studies did not calculate the PI, the PI calculation of 2 studies did not meet the inclusion criteria. After initial eliminations, 16 studies appeared to meet inclusion/exclusion criteria. Among the included studies, 6 [25]–[30] studies were found to share common sample sources with 3 other studies [31]–[33]. Those studies with largest sample size were used in our meta-analysis. 2 studies [23], [34] did not provide standard deviation or mean PI. Finally, 8 articles in total [15], [31]–[33], [35]–[38] were accepted for our meta-analysis. The reference lists did not provide any additional articles. A total of 7 of the included studies [15], [31]–[33], [35]–[37] had reported the Pearson correlation coefficient between PI and MnB (Figure 1).

### Study characteristics

8 articles were identified as meeting the inclusion criteria. There were 281 cases of occupationally Mn-exposed workers. The research on occupationally Mn-exposed group came from Germany, Korea, China, South Africa and the United States, while the research on the chronic liver disease group came mainly from Japan, Canada, Korea and China. The minimum average age of the subjects of the included studies was 9 years old and the maximum was 56.2 years old. All of the included studies were retrospective studies and used over 0.5T MRI to record measurements of brain Mn accumulation. The experimental subjects were smelters and welders. Of the included studies, 7 studies reported the Pearson correlation coefficient of PI and MnB (n = 255) (Table 2).

**Table 2:** Characteristics of included studies.

Study	Country	Study design	MRI mode	Sample size Exp/Ct (n)	Objects (Exp/Ct)	Means of PI (Exp/Ct)	S.D. of PI (Exp/Ct)	Pearson correlation coefficient	Number of correlation analysis	Average age(year)
Kim et al. 1999, [15]	Korea	RS	1.0T MRI	89/32	Welder, smelter	107.7/103.9	6.5/3.3	0.410	89	43.3
Dietz et al. 2001, [35]	Germany	RS	0.5 T MRI	8/10	Dry battery factory workers	93.4/90.8	1.1/2.7	0.480	8	40
Choi et al. 2007, [33]	Korea	RS	1.5T MRI	20/10	Welder	112.0/106.0	4.0/3.0	0.350	30	44.3
Jiang et al. 2007, [32]	China	RS	1.5T MRI	18/9	Smelter	116.2/102.2	8.4/1.5	0.090	18	35.8
Chang et al. 2009, [31]	Korea	RS	3.0 T MRI	43/29	Welder	124.1/117.2	11.2/3.4	0.510	43	48.9
Li et al. 2010, [36]	China	RS	3.0T MRI	18/19	Smelter	116/101	9.0/5.0	0.200	18	41
Kim et al. 2011, [37]	Korea	RS	3.0 T MRI	30/19	Welder	124.6/116.7	11.9/2.5	0.557	49	48.3
Nelson et al. 2012, [38]	South African	RS	3.0T MRI	13/10	Mn workers	124.2/114.7	11.2/7.2	N/A	N/A	39

Note: RS, Exp/Ct and S.D. represent retrospective study, experimental group/control group, and standard deviation, respectively.

### The meta-analysis results of PI

**Figure 2:**
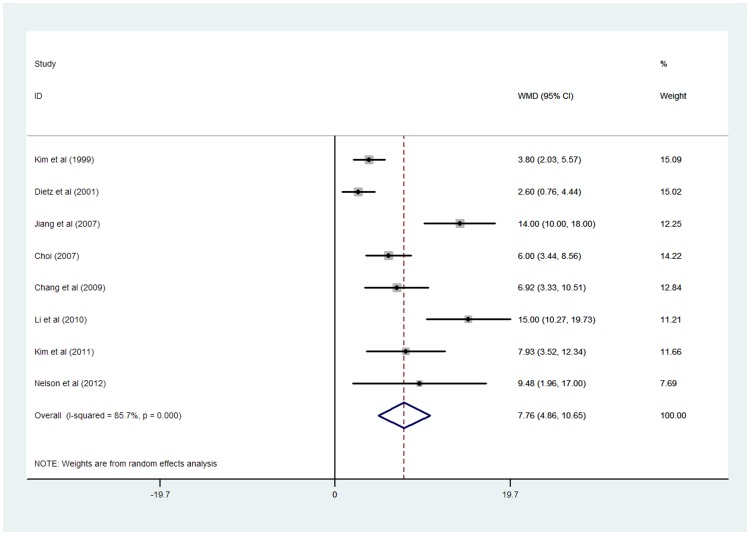
The forest plot of the PI in the experimental group comparing with the control group.

The heterogeneity statistic (*I^2^)* values (*I^2^* = 85.7%, *p*<0.0001) was observed to be more than 50%; using the random effect model, meta-analysis showed that the WMD combined effect of PI is 7.76 (95% CI 4.86, 10.65). The result of the combined effect test (Z = 5.25, *p*<0.0001) showed that there was significant difference in the PI between the control and experimental groups. In the forest plot, the horizontal line skewed to the right, indicating that the PI of the experimental group was higher than that of the control group (Figure 2).

### Test of heterogeneity

The heterogeneity was very high. Therefore, we performed a meta-regression analysis to identify the sources of heterogeneity by country, experimental group subjects, MRI models, and average age. However, when we categorized the heterogeneity by these factors none significantly contributed to the observed heterogeneity.

### Sensitivity analysis

Sensitivity analysis was performed by the sequential removal of individual studies and cumulative statistics for all comparisons of all subjects showed that the summary results did not change significantly, indicating good stability of the included studies.

### Publication bias

Funnel plots and Begg's test were performed to assess publication bias. The data suggested that there was publication bias of the included studies (Begg's test *p* = 0.063, Egger's test *p* = 0.014).

### Meta-analysis results of PI–MnB relationship

**Figure 3:**
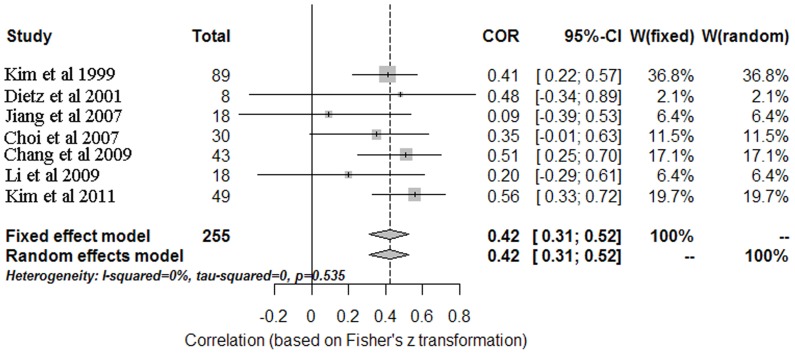
The forest plot of the PI–MnB relationship.

The result of the heterogeneity statistic in both of groups showed no heterogeneity between the included studies (*I^2^* = 0%, *p* = 0.535), which indicated that the most appropriate pooling model is the fixed effect model. A fixed effect meta-analysis included 7 studies. The strength of subjects ascribing PI was correlated with MnB (r = 0.42; 95% CI, 0.31, 0.52; Z = 6.91, *p*<0.0001; Figure 3).

## Discussion

Cumulative studies have been devoted to investigate the underlying mechanisms of the extrapyramidal neurological damage caused by Mn. It is certain that Mn can pass through the blood-brain barrier and accumulate in the brain, and it is a paramagnetic material which can shorten the T1 relaxation times(T1-RT) and symmetrically enhance the signal intense in the Mn accumulation target, GP [39]–[41]. Mn can primarily and selectively accumulate in the GP, followed by deposition in the substantia nigra, striatum, pineal gland, olfactory bulb and substantia nigra pars compacta [42]–[44]. The autopsy results from patients with cirrhosis showed significantly higher Mn levels in the GP, caudate nucleus, substantia nigra and ventral tegmental area of the midbrain than those of controls [22]. Compared with normal signal intensity patients, the Mn level in GP was accompanied by signal enhancement of MRI T_1_-WI by up to10 times in GP of cirrhosis patients. These results suggested that GP T_1_-WI signal enhancement was mainly due to the accumulation of Mn in GP [45]–[47]. Furthermore, the characteristic high signals were also frequently observed in experimental Mn poisoning of non-human primates [14], [48]–[50], asymptomatic workers exposed to Mn [15], [31], [32], [34] and manganism patients [51]. The consistency of areas of brain Mn accumulation and areas of MRI T_1_-WI high signal intensity indicate that it is sensitive and specific for MRI positioning brain Mn accumulation.

Nowadays, there are a few methods applied to detect Mn levels in humans, including analysis of Mn levels in teeth [52], urine [53] and hair [54], [55], NMR T_1_ measure proton relaxation times of local GP [7], serum or whole blood Mn levels measured by atomic absorption spectrometry [56];MRI depicted brain Mn deposition [57], etc. However, MRI examination is the best method for obtaining information on brain anatomy, function and metabolism [58]. PI measurement has been widely used to estimate tissue Mn level in the brain due to its simplicity of measurement [59], [60]. In addition, PI has a longer half-life than MnB and is a better predictor of neurobehavioral performances [31]. Furthermore, PI showed a significant dose-response relationship with MnB in Mn-exposed non-human primates [49], patients with manganism, and asymptomatic Mn-exposed workers [31].

However, issues still exist regarding using PI as a biomarker to diagnose the early neurotoxic effects of Mn. Previous studies suggested that PI might be less sensitive to lower, subclinical levels of Mn exposure than other brain tissue (e.g., Olfactory Bulbs) [34], [49]. Choi et al [33] found that using PI as a semi-quantitative index of target site dose was limited over a narrow range of Mn level due to the non-linear nature of its dose–response relationship. Nevertheless, studies also showed that PI has strong correlations with environmental Mn exposure levels, exposure hours, and neurobehavioral performances [25], [37], [60], [61]. The small sample sizes, races and the wide ranges of variance of MR indices may create those inconsistent phenomenons. Our meta-analysis substantially approved that MRI technology has specificity in detecting brain Mn accumulation caused by occupationally Mn-exposed. The PI of the experimental group was higher than that of the control, suggesting that PI could semi-quantitatively reflects MRI T_1_-WI signal enhancement which was valuable for the detection of brain Mn deposition. In addition, the PI–MnB relationship in meta-analysis showed that the PI correlated strongly with MnB levels further confirmed the significance of PI in evaluating Mn levels in the brain, and MnB may also be used to strengthen this evaluation.

### Heterogeneity

Previous study stated that it was difficult to avoid heterogeneity in meta-analysis [62]. With regard to our studies, we mentioned several problems that may be responsible for heterogeneity: a. Different geographical locations of the included studies: six studies were from East Asia (China and Korea), while others were from Germany and South Africa; b. MRI models: MRI models in all of the included studies were over 1.0T MRI models, while one study used a 0.5T MRI model; c. Ascertainment of experimental group subjects: one of the experimental groups from one of the included studies in the occupationally Mn-exposed group came from dry battery factory workers, while others came from welders or smelters; d. Different sample sizes: one of the included studies [35] had small sample sizes below 10.

### Limitations

Results from our meta-analysis must be viewed cautiously due to its own limitations. Firstly, we only selected English and Chinese published or unpublished literature, thus linguistic bias exists. Secondly, given the limitation of local libraries and hospitals we only selected the literature for which original full-text was likely to be found, which would cause selection bias. Thirdly, PI may be affected by pulse sequences as well as Mn exposure. Last but not least, there were publication bias and large statistical heterogeneity could be found among our included studies, which might induce the existence of bias factors. Despite only identifying 8 studies for PI meta-analysis and 7 studies for PI and MnB correlation meta-analysis, which used different MRI models, unequal sample sizes and different subjects of experimental groups; as the original data were not available for further analysis, the results should be interpreted with caution. In spite of these limitations, we have tried to moderate and explain them.

### Summary

In summary, our results showed that PI of the experimental groups was higher than those of the controls and there were significant correlations between PI and MnB levels confirmed that PI is a sensitive, reliable and effective semi-quantitative indicator to evaluate the Mn accumulation levels in the “target organ” (brain tissue) and “target” (GP). It can be used as a biomarker to diagnose the early neurotoxic effects of Mn.

## Supporting Information

Checklist S1 **PRISMA Checklist.**
(DOC)
